# Membraneless organelles: a smart design for metabolic control

**DOI:** 10.1002/2211-5463.13264

**Published:** 2021-09-01

**Authors:** Irene Díaz‐Moreno, Miguel A. De la Rosa

**Affiliations:** ^1^ Instituto de Investigaciones Químicas Universidad de Sevilla‐CSIC Spain

**Keywords:** cytochrome *c*, liquid‐liquid phase separation, membraneless organelles, polyproline II helices, prion‐like proteins

## Abstract

While most organelles are surrounded by membranes, cells also contain membraneless organelles, which remain separated in the cell by avoiding the mixture of their components with the surroundings. Actually, liquid–liquid phase separation provides a simple but smart mechanism for the cell to control the spatial localization and processing of molecules, without relying on membrane boundaries. This Special ‘In the Limelight’ section, entitled ‘Membraneless organelles’, consists of three review articles, each focused on a particular aspect. The first article deals with assembly of coacervates as mediated by polyproline II helices, as well as with condensate stability. The second article addresses the formation of protein–nucleic acid coacervates by prion‐like proteins and their link to human diseases. Finally, the last article focuses on mitochondrial cytochrome *c* translocation into the nucleus after DNA damage, with the subsequent inhibition of nucleosome assembly/disassembly activity of histone chaperones and its impact on chromatin dynamics and nuclear condensates.

## Graphical abstract

This Special ‘In the Limelight’ section consists of three review articles on aspects related to membraneless organelles. The first article deals with coacervate assembly as mediated by polyproline II helices, the second addresses the formation of protein–nucleic acid coacervates by prion‐like proteins, and the last article focuses on mitochondrial cytochrome *c* translocation into the nucleus after DNA damage.

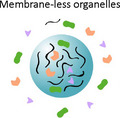

**Abbreviations**
MLOs: membraneless organelles
PPII: polyproline II

How do cells enable internal spatiotemporal control of complex biochemical reactions? They have partly solved this question by creating compartments, or organelles, that allow distinct chemical environments. Most of these compartments are surrounded by membranes (nucleus, mitochondria, chloroplasts, lysosomes, etc.). It is thus easy to understand how such membrane‐bound compartments cohabit and work in the cells. On the contrary, many other compartments (nucleoli, centrosomes, Cajal bodies, stress granules, etc.) lack membranes, making their coexistence inside cells harder to explain. Such membraneless organelles (MLOs) remain separated in the cell by avoiding mixture of their components with the surroundings. The following questions soon arise: ‘Are there molecules transported in and out of MLOs?’ and ‘How fast must the diffusion of the components within MLOs be to guarantee efficient chemical reactions?’.

Recent experimental evidence has shown that many MLOs are liquid droplets formed by phase separation, thereby allowing MLO components to be rapidly concentrated in a concrete place in the cell. The transient assembly of liquid drops is driven by (a) multivalent weak interactions between signalling domains repetitively included in proteins and RNA and (b) substantial conformational heterogeneities of intrinsically disordered regions. In the case of cell pathology and devastating aggregation diseases, liquid–liquid demixing can eventually result in metastable condensates of intracellular matter, such as glass/hydrogels or amyloid‐like fibres, that undergo liquid–solid phase transitions [1, 2]. Under normal conditions, liquid–liquid phase separation provides a simple but smart mechanism for the cell to control the spatial localization and processing of molecules, without relying on membrane boundaries.

The three review articles in this Special ‘In the Limelight’ section, entitled ‘Membraneless organelles’, are focussed on how such nonequilibrium events in living cells control intracellular phase behaviour. It is noteworthy that different types of biomolecular condensates do not generally combine to form larger microdroplets, thereby suggesting that each of them uses a distinct class of attractive interactions. Within this frame, Laurents and coworkers discuss in the first article whether polyproline II (PPII) helices mediate the assembly of coacervates, as well as if the condensate stability is influenced by either the number of PPII helical tracts or the tandem PPII‐binding domains [3]. The potential, new role of PPII could broaden the palette of verified interactions contributing to biomolecular condensate formation. Prion‐like proteins are the main topic addressed by Ventura and coworkers in the second article [4]. Interestingly enough, prion‐like proteins are not only connected with the formation of functional membraneless protein–nucleic acid coacervates, but are also linked to human diseases, including cancer, neurodegenerative disorders and viral infections. In the last article, De la Rosa and coworkers discuss their recent description of the translocation of mitochondrial cytochrome *c* into the nucleus after DNA damage, where the hemeprotein inhibits the nucleosome assembly/disassembly activity of histone chaperones by binding to their acidic disordered regions [5]. Such interaction impacts on chromatin dynamics and nuclear condensates, including heterochromatin, by impairing heterotypic contacts between chaperone acidic stretches and histone Lys‐rich tails, which are both responsible for liquid–liquid phase transitions.

We would like to thank all of the authors for their excellent contributions to this ‘In the Limelight’ section and hope the provided insights into this fascinating field will be of interest to all of our readership.

## Conflict of interest

The authors declare no conflict of interest.
